# CLABSIs aren’t just for inpatients: the need to identify CLABSI burden among outpatients

**DOI:** 10.1017/ash.2024.384

**Published:** 2024-09-12

**Authors:** Opeyemi Oladapo-Shittu, Sara E. Cosgrove, Clare Rock, Yea-Jen Hsu, Eili Klein, Anthony D. Harris, Carlos Mejia Chew, Heather Saunders, Patrick R. Ching, Avi Gadala, Stephanie Mayoryk, Lisa Pineles, Lisa L. Maragakis, Alejandra B. Salinas, Taylor Helsel, Sara C. Keller, for the Centers for Disease Control and Prevention’s Prevention Epicenters Program

**Affiliations:** 1 Johns Hopkins University School of Medicine, Baltimore, MD, USA; 2 Johns Hopkins Bloomberg School of Public Health, Baltimore, MD, USA; 3 University of Maryland School of Medicine, Baltimore, MD, USA; 4 University of Maryland School of Public Health, Baltimore, MD, USA; 5 Washington University School of Medicine, St. Louis, MO, USA; 6 Virginia Commonwealth University School of Medicine, Richmond, VA, USA

National estimates of central line-associated bloodstream infections (CLABSI) are likely underestimated because only those that occur more than 72 hours after admission to an acute care hospital are routinely reported to the Centers for Disease Control and Prevention’s (CDC) National Healthcare Safety Network (NHSN).^1^ However, central lines (CLs) are increasingly used outside of acute care hospitals,^2^ in locations such as the patient home, outpatient infusion or chemotherapy centers, skilled nursing or long-term care facilities, home-based or facility-based dialysis centers, and rehabilitation facilities. CLABSIs that arise in these settings are not included in surveillance or reported systematically. Furthermore, patients often transition between patient homes, outpatient infusion centers, chemotherapy centers, skilled nursing or long-term care facilities, dialysis centers, rehabilitation facilities, and acute care hospitals (Figure 1), making it difficult to standardize infection tracking and reporting. Knowing the burden of CLABSI outside of the hospital (community-onset CLABSI, or co-CLABSI) and the locations in which they develop is essential for understanding how to develop and deploy CLABSI prevention resources. In this viewpoint, we discuss several issues regarding co-CLABSI including their patient-level impact, prevalence estimates, and challenges with identification and infection rate calculation in community settings.

**Figure 1.**
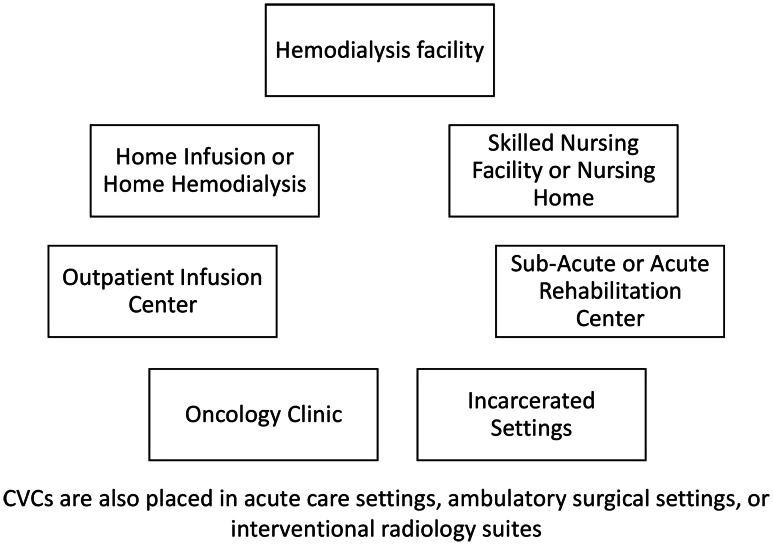
Location of central venous catheter placement and maintenance in the community.

## Impact of co-CLABSI

Co-CLABSIs can lead to significant morbidity and mortality: among patients presenting to the hospital with co-CLABSI, 25% required intensive care and 11% died in the hospital.^3^ Furthermore, patients with CLs in community settings often require the CL for extended periods, for purposes such as hemodialysis when an arteriovenous graft or fistula is not an option, long-term parenteral nutrition in patients without a functioning gastrointestinal tract, or chemotherapy. In addition to infection-related morbidity, CL removal due to CLABSI can result in additional complications including treatment delays and challenges in securing new parenteral access.^4,5^

## Current systematic data available regarding co-CLABSIs

Due to a lack of a surveillance system for co-CLABSI, the frequency of co-CLABSI is generally not measured. However, some community healthcare locations managing patients with CLs, such as dialysis centers, collect limited data on the frequency of overall bloodstream infection (BSI). Dialysis centers report dialysis events, which include but do not differentiate CLABSIs from other BSIs, including those in patients with arteriovenous fistulas or arteriovenous grafts, localized infections of vascular access sites, or any intravenous antimicrobial start.^6^ Furthermore, dialysis events are underreported—only 11% of dialysis events involving methicillin-resistant Staphylococcus aureus are reported to the NHSN.^7^

Recent work has successfully implemented an adapted CLABSI definition for home infusion therapy on a small scale,^8^ but this definition has not yet been widely adopted. In addition, there is currently no structure or requirement to report home infusion CLABSI to accrediting organizations or to the NHSN, making it difficult to know what CLABSI rates are outside of voluntary data collaboratives.^9^ Long-term care facilities and outpatient infusion or chemotherapy centers are not required to report CLABSIs to the NHSN, and large-scale investigations of CLABSI rates in these settings are lacking, with the exception of academic pediatric oncology centers.^10–16^

## A proposed approach to quantify co-CLABSI

In the absence of many community healthcare locations reporting CLABSI, alternative approaches to measure the number of co-CLABSI are needed. Acute care hospitals could coordinate with community locations and help these locations identify when a patient is admitted with a CLABSI. Most patients with co-CLABSI likely are admitted to an acute care hospital for evaluation and treatment. Assessing CLABSI-POA present at the time of hospital admission (POA) or occurring within the first 72 hours of hospital admission in patients admitted with CLs from the community could provide clues to the burden of co-CLABSI. A single-hospital study suggested that there were more CLABSI-POA in that hospital than acute care CLABSI over 1 year (130 CLABSI-POA vs 90 hospital-onset CLABSI).^17^ Separately, we identified 461 CLABSI-POA in 11 hospitals in 3 health systems over the course of a year.^18^ This approach can approximate co-CLABSI but does not provide a rate or allow calculation of the incidence.

As an added benefit, assessing CLABSI-POA could assist in providing feedback to community healthcare locations where CLs are maintained. Currently, without a clear reporting mechanism and requirement, it is difficult for a community-based healthcare organization to identify when a patient they have been following has been admitted and diagnosed as having CLABSI.^19^ Community-based healthcare organizations such as home infusion agencies must put significant effort into an investigation when a patient has been admitted with CLABSI. To reduce the burden on community-based healthcare sites that generally have minimal infection prevention resources, acute care hospital infection prevention and healthcare epidemiology teams could measure CLABSI-POA and report these back to community healthcare organizations.^19,20^ Having a strategy to communicate back to the community-based healthcare team when a patient is admitted to an acute care hospital with a co-CLABSI is an essential part of reducing the co-CLABSI burden. Acute care communication with the community healthcare organization may help increase the organization’s awareness of CLABSI risk and prompt implementation of CLABSI prevention interventions.

## Approaches to calculating co-CLABSI rates

To calculate co-CLABSI rates, it is necessary to have both a numerator and a denominator. To develop an appropriate denominator for the community setting, it is necessary to know how many patients with CLs are in the community and, ideally, the amount of time they have CLs in place (Table 1). Having a centralized registry of patients with CLs in communities would aid in developing an accurate denominator, but no such registry exists. Those developing a CL registry would need to consider that CLs are placed, maintained, and accessed in multiple different care locations, and an individual patient may transition between multiple care sites and regions. A potential approach could be through maintaining a list of CLs that were placed in ambulatory settings (eg, with specific procedure codes in an ambulatory interventional radiology suite). Such a registry would require significant resources to implement but would be useful for quantifying co-CLABSI and monitoring the success of co-CLABSI prevention efforts. For example, healthcare sites often do not share staff or a common medical record; thus, tracking the presence of a CL after a patient is discharged from the hospital is challenging. In addition, it is not standard practice to record CL removals in medical record systems outside of acute care hospitals. Therefore, any list of CLs placed in ambulatory settings could quickly become inaccurate if removals were not recorded. A mandate to better share data on CLs through a dashboard or health information exchange^21^ would be necessary to track CLs in the community.

**Table 1.** Barriers to CLABSI surveillance outside of acute care hospitals

Barrier	Potential mitigating strategy
No centralized registry of CLs	Use EHR tools to monitor CLs in a standardized way across health systems and healthcare providers
Multiple locations where CLs are placed	Require all locations where CLs are placed (including those in ambulatory settings) to document in a shared EHR
Multiple care locations	Encourage the use of a shared EHR; implement a standardized way to document and track CLs outside hospitals including regional HIE networks
Multiple care transitions	Develop EHR-based approaches that follow an individual CL (and not just an admission) over time; include different locations in verifying information about the CL
Unclear when CLs are removed	Encourage and facilitate efforts to document in an EHR when CLs are removed
No standard approach to tracking CLs	Use regional HIE networks as a backup to document information about CLs; encourage all organizations covered by an HIE to document in the HIE
Port access timing is unclear	Develop different approaches to follow ports for CLABSI surveillance purposes in the community, such as including these in a denominator if there is access over a month

Note. EHR, electronic health records; CL, central line; CLABSI, central line-associated bloodstream infection; HIE, health information exchange.

CLs that are most frequently used in ambulatory settings include tunneled dialysis catheters, tunneled CLs, pheresis catheters, peripherally inserted central catheters, and ports.^22^ Ports may cause particular difficulties in calculating CL days in ambulatory settings as they are so commonly used in the home, outpatient clinics, and oncology settings.^23^ In acute care hospitals, a port must be accessed for over 2 days to become eligible for CLABSI reporting and contribute to CL days for the rest of a hospitalization.^24^ However, outside of hospitals, the frequency of port access varies significantly for patients. Although some patients access ports daily for continuous access and infusions, others may only access their ports every 1–2 months for routine maintenance (ie, flushing)^25^ or not access a port for years. Knowing when a port is accessed or de-accessed for purposes of determining when a patient with a port might be eligible for a CLABSI can be difficult in ambulatory settings as this data is not commonly captured. Yet, no standard exists for when ports should be considered eligible for CLABSI reporting in the ambulatory setting. Our view is that ports should be eligible for CLABSIs if they were accessed within the last month, and all calendar days in a month during each month in which the port was accessed should be included in CL days for calculating co-CLABSI rates.^8^ These data would need to be recorded and captured in some way to facilitate CLABSI surveillance.

## Summary

CLABSIs in the community may be underrecognized and may cause significant morbidity and mortality. Acute care hospital infection preventionists have the expertise to collaborate with community providers to identify and prevent co-CLABSI but may themselves require additional support to share this expertise. Mandatory reporting measures and resulting reimbursement models would prompt more organizations to collaborate on co-CLABSI prevention. An increased regulatory emphasis on healthcare-associated infection (HAI) prevention at care transitions could help with starting to build the infrastructure around co-CLABSI reporting. However, to understand the burden of disease, it is important to understand the number and rates of co-CLABSI. State health departments should develop ways to track health devices to better understand the number of devices in the community, such as through a dashboard or health information exchange tool, and work closely with other community-based health providers to provide feedback to community-based health providers about complications and rates. Additional resources and infrastructure would be needed to assist with education and devoted time for surveillance^26^ but may be difficult to access in the current funding climate. This could be made easier with simplified surveillance definitions. As health care continues to transition to the ambulatory setting, monitoring for HAIs in ambulatory care is increasingly important.
